# Technology Assisted Rehabilitation Patient Perception Questionnaire (TARPP-Q): development and implementation of an instrument to evaluate patients’ perception during training

**DOI:** 10.1186/s12984-023-01146-3

**Published:** 2023-03-24

**Authors:** Cira Fundarò, Roberto Casale, Roberto Maestri, Silvia Traversoni, Roberto Colombo, Silvana Salvini, Chiara Ferretti, Michelangelo Bartolo, Michelangelo Buonocore, Anna Giardini

**Affiliations:** 1 Istituti Clinici Scientifici Maugeri Spa SB IRCCS Neurophysiopathology Unit of Montescano Institute, Pavia, PV Italy; 2 OPUSMedica PC&R, Persons, Care & Research, Piacenza, Italy; 3 Istituti Clinici Scientifici Maugeri, IRCCS Department of Biomedical Engineering of Montescano Institute, Pavia, PV Italy; 4 Istituti Clinici Scientifici Maugeri IT Department, IRCCS Pavia, Pavia, PV Italy; 5 Istituti Clinici Scientifici Maugeri IRCCS Veruno, Veruno, NO Italy; 6 Istituti Clinici Scientifici Maugeri IRCSS Neuromotor Rehabilitation Unit of Montescano Institute, Pavia, PV Italy; 7 Habilita Department of Rehabilitation, Neurorehabilitation Unit, HABILITA Zingonia, Ciserano, Bergamo, Italy

**Keywords:** Technology-assisted rehabilitation, Exergaming, Augmented performance feedback, Self-report, Patient experience, Questionnaire

## Abstract

**Background:**

The introduction of technology-assisted rehabilitation (TAR) uncovers promising challenges for the treatment of motor disorders, particularly if combined with exergaming. Patients with neurological diseases have proved to benefit from TAR, improving their performance in several activities. However, the subjective perception of the device has never been fully addressed, being a conditioning factor for its use. The aims of the study were: (a) to develop a questionnaire on patients’ personal experience with TAR and exergames in a real-world clinical setting; (b) to administer the questionnaire to a pilot group of neurologic patients to assess its feasibility and statistical properties.

**Methods:**

A self-administrable and close-ended questionnaire, Technology Assisted Rehabilitation Patient Perception Questionnaire (TARPP-Q), designed by a multidisciplinary team, was developed in Italian through a Delphi procedure. An English translation has been developed with consensus, for understandability purposes. The ultimate version of the questionnaire was constituted of 10 questions (5 with multiple answers), totalling 29 items, exploring the patient’s performance and personal experience with TAR with Augmented Performance Feedback. TARPP-Q was then administered pre-post training in an observational, feasible, multi-centric study. The study involved in-patients aged between 18 and 85 with neurological diseases, admitted for rehabilitation with TAR (upper limb or gait). FIM scale was run to control functional performance.

**Results:**

Forty-four patients were included in the study. All patients answered the TARPP-Q autonomously. There were no unaccounted answers. Exploratory factor analyses identified 4 factors: Positive attitude, Usability, Hindrance perception, and Distress. Internal consistency was measured at T0. The values of Cronbach’s alpha ranged from 0.72 (Distress) to 0.92 (Positive attitude). Functional Independence Measure (FIM®) scores and all TARPP-Q factors (Positive attitude, Usability, Hindrance perception, except for Distress (p = 0.11), significantly improved at the end of the treatment. A significant positive correlation between Positive attitude and Usability was also recorded.

**Conclusions:**

The TARPP-Q highlights the importance of patients’ personal experience with TAR and exergaming. Large-scale applications of this questionnaire may clarify the role of patients’ perception of training effectiveness, helping to customize devices and interventions.

**Supplementary Information:**

The online version contains supplementary material available at 10.1186/s12984-023-01146-3.

## Introduction

Neuro-motor rehabilitation is defined as a problem-solving process focused on the betterment of a patient’s functional activities and aiming to improve both motor outcomes and quality of life [1]. Its effectiveness generally depends upon several heterogeneous factors, ranging from the training protocol to the patient’s characteristics [2, 3] and to external variables, such as the relationship between patient and health care professional or the care setting [4, 5].

In the last decades, the advances in medical technology fuelled the transition from front-to-front human treatment to technology-assisted training [6]. Rehabilitation technology has, indeed, witnessed an increasing succession of high-tech implementations in real-life settings, such as wearable devices, and robotic devices integrated with exergaming [7, 8], including Virtual Reality (VR) interfaces and Augmented Feedback modules [7, 9].

These latter developments are designed to integrate serious games into common rehabilitation tasks, empowering the therapist’s efficiency, patients’ motivation, adherence, and motor recovery [7, 10–12].

Besides, when it comes to practice, it is pivotal to ensure patients' comfort and well-being while approaching innovative yet unfamiliar devices [4, 8, 13].

Hence, research on rehabilitation technology is called to develop sound and validated methods to assess technology-assisted devices in terms of safety, end-user degrees of acceptance, adherence, and satisfaction [14–17]. Recent reviews reported how different and heterogeneous assessment tools were often used for the evaluation of Technology-Assisted Rehabilitation (TAR) [18, 19]*.* Furthermore, in previous works, patient-device interaction was assessed considering single and very specific issues, such as the patient’s emotional state [10, 11, 16, 17, 20], motivation [12, 21–23], psychosocial impact [24] and usability [25, 26] of the device adopted. In this regard, a limited number of instruments have already been applied [26–29], pertaining few distinct aspects of patient-device interaction [29]. As a result, according to our review, the current lack of a dedicated scale integrating the multiple features of TAR limits the comparison of findings regarding their effectiveness.

The aims of the present study are: (a) to develop a questionnaire on patients’ experience with rehabilitation and technology-assisted devices in a real-world clinical setting; (b) to administer the questionnaire to a pilot group of neurologic patients to assess its feasibility of administration and statistical properties.

## Methods

### Study design

The study was composed of two subsequent phases: questionnaire development through Delphi methodology and questionnaire application. In the second phase, an observational, feasible, multi-centric study relating to patients’ experiences with TAR was conducted in the Neurorehabilitation Unit of Istituti Clinici Scientifici Maugeri Spa SB- IRCCS Montescano, Italy, and at the Habilita Zingonia Centre of Ciserano, Italy.

### Ethics approval and consent to participate

The study design and the protocol were submitted and approved by the Institutional Review Board and by the Ethics Committee (Comitato Etico Istituti Clinici Scientifici ICS Maugeri Pavia, approval CE number 2206 date 29.5.2018) and were implemented following the World Medical Association code of Ethics (Declaration of Helsinki, 1967).

### Questionnaire development

A multidisciplinary team (neurologists, psychologists, bioengineers, physiatrists, and rehabilitation therapists specialized in technological devices for rehabilitation) was constituted to specifically work on the creation of the Technology Assisted Rehabilitation Patient Perception Questionnaire (TARPP-Q). The working board aimed to create a multidimensional, self-administrable, and close–ended questionnaire to evaluate the different facets of patient experience with TAR devices and exergaming.

**Fig. 1:**
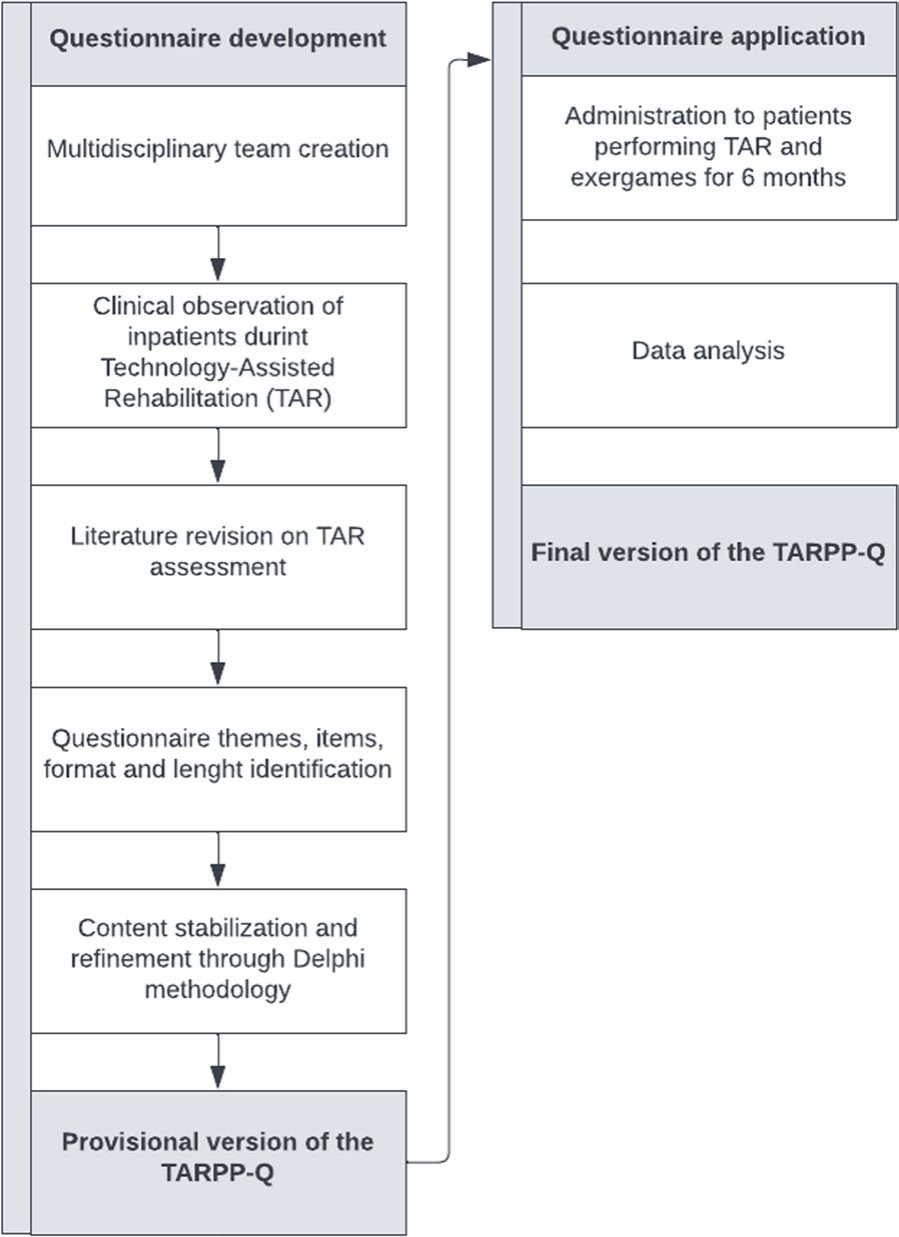
Questionnaire construction process

The process followed a bottom-up approach started by freely observing inpatients during high-technology training; clinical observations prompted a literature revision on the development of questionnaires administered to assess patients’ experience with TAR. Based both on the existing literature and on clinical experience, the team independently and freely identified a pool of items that were then submitted to a Delphi methodology and progressively reviewed to reach a general agreement [30] (Fig. 1 Questionnaire construction process). The same procedure was applied to determine the questionnaire format, items’ number, questionnaire length, and rating scale degree*.*

The questionnaire was developed and administered in Italian and subsequently translated into English.

The full version of the questionnaire in English is provided for understandability purposes. Three independent investigators (AG, ST, RCa), whose native language was Italian, translated the original version of the questionnaire into English. An idiomatic translation was preferred over a word-for-word transposition. The investigators compared the 3 translated versions, with consensus. Lastly, an independent translator who had not participated in the first stage and whose native language was English supervised and finalized the last translation, with consensus.

### Questionnaire application

The first version of the TARPP-Q, obtained through Delphi consensus, was administered over 6 months to consecutively admitted inpatients. Patients underwent rehabilitation training with TAR and exergaming at the third (T0) and the last training session (T1).

### Sample

Inclusion criteria: in-patients, regardless of gender, aged between 18 and 85 years of age, admitted for a TAR program with exergaming due to a neurologic disease.

Exclusion criteria: cognitive deficits (MMSE ≤ 22) [31], insufficient knowledge of written or spoken Italian language, aphasia, severe visual or auditory deficits.

### Data collection

The following data were collected at admission: age, years of education, type of disease, and disease duration. In addition**,** the FIM scale [32] was administered at T0 and T1. Patients were requested to give their informed consent to the study and the authorization of scientific treatment of their medical records in an anonymous form.

### Technology-assisted training

According to their prevailing functional deficit, patients were subjected to a daily upper limb (effective 30-min) technology-assisted treatment or to a daily (30-min) technology-assisted gait training for 5 days/week for 4 weeks. The first and the second session were for adaptation. There was no break during gait session training, while there was a 1-min break between one exercise and another to select the following exercise by the operator during upper limb rehabilitation.

The training was conducted with exergames integrated with the upper-limb and gait devices. The exergames of the two devices used are defined by the vendor (Hocoma, Switzerland) as Augmented Performance Feedback (APF) activities. More specifically, the devices present an inbuilt software that can be used to enhance motivation by providing visual and interactive screen feedback on the ongoing motor performance. The active and live effort from the user is therefore represented on a display monitor (scores, accuracy rates, etc.) through different available games.

**Fig. 2:**
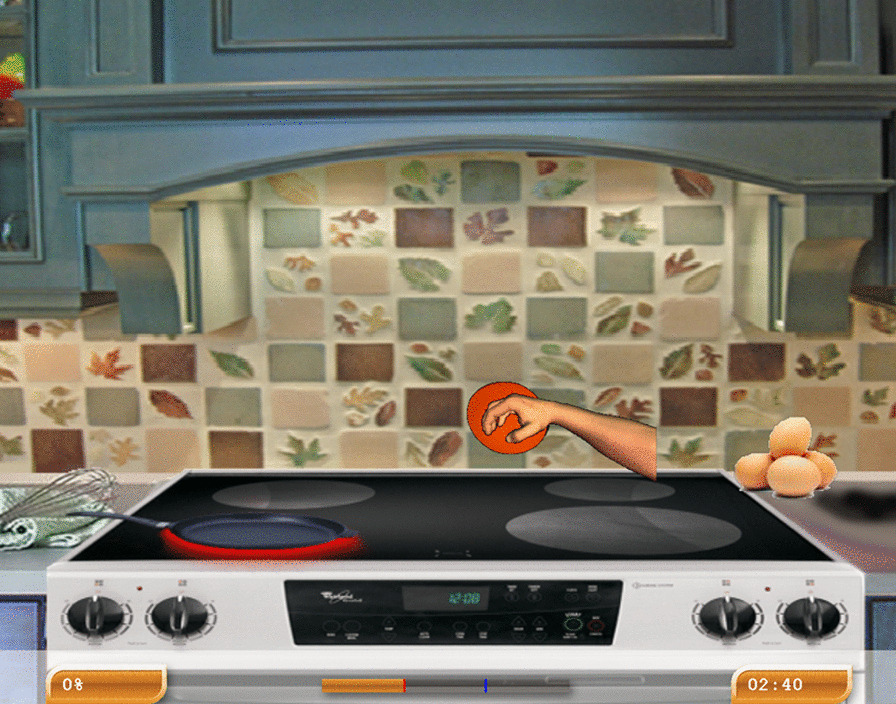
Armeo’s exercise example 1

**Fig. 3:**
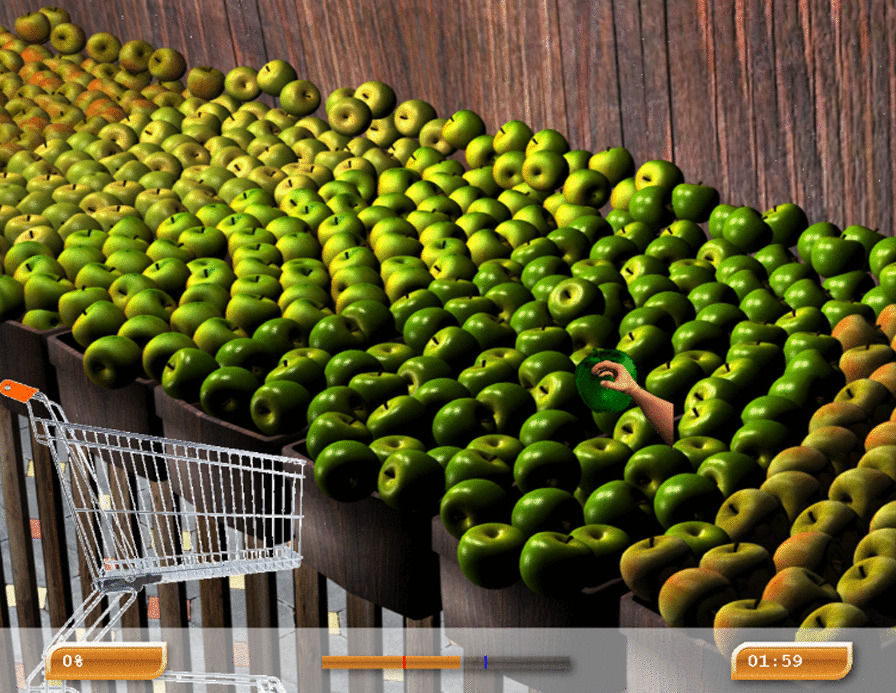
Armeo’s exercise example 2

**Fig. 4:**
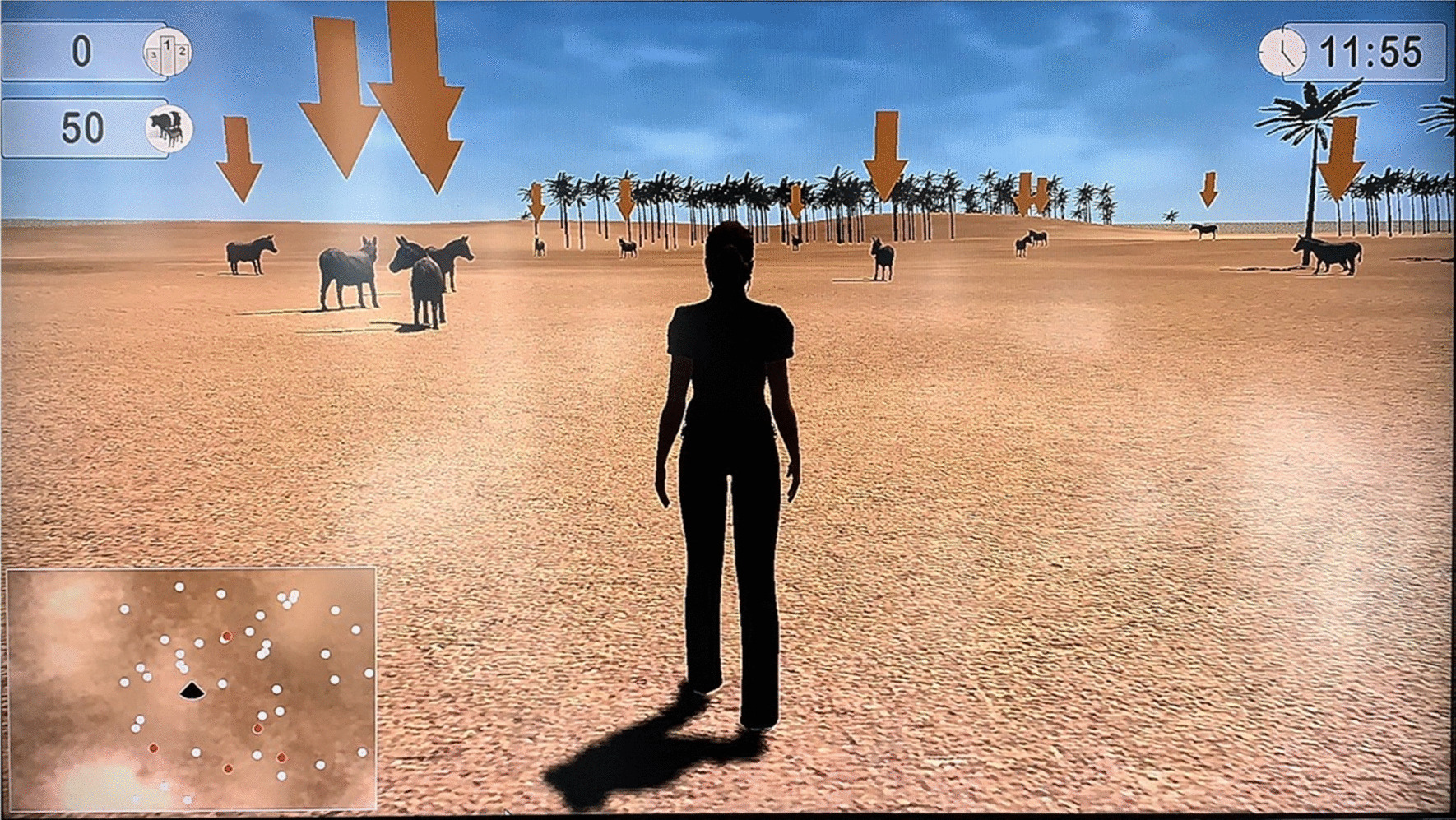
Lokomat’s exercise example

Three examples of exercises were below detailed (Figs. 2, 3, 4).

### Upper limb rehabilitation

Upper limb-assisted training was performed by Armeo Spring ® (Hocoma, Switzerland), consisting of mechanical anti-gravity support equipped with 8 joints that permit movement in three-dimensional space; in addition, it is provided with a handle with which the patient can execute a grab gesture, thus allowing for the evaluation of the grip force. The ArmeoSpring ® provides real-life exergaming with APF, partially simulated activities of daily living*,* to conduct therapeutic exercises applicable in a safe environment. Namely, patients actively perform training in front of a screen, interacting with a semi-immersive environment.

### Gait rehabilitation

Technology-assisted gait training was performed by Lokomat® (Hocoma, Switzerland) constituted by an exoskeleton, a body weight support, and a treadmill. It facilitates symmetric hip and knee gait patterns thanks to its exoskeleton, driven by programmable actuators.

Rehabilitative exercises with APF are carried out in a semi-immersive environment. During all training sessions, patients were placed in front of a screen where a representation of the patient simulated walking in a virtual landscape, guided by the patient’s movements.

### Functional Independence Measure (FIM®)

The Functional Independence Measure (FIM®) scale measures the patient's disability level and indicates the degree of assistance required for the subject to carry out activities of daily living; it is largely adopted in rehabilitation as a functional outcome indicator [32]. It consists of 18 items, considering motor (13 items) and cognitive (5 items) functions, respectively. Each FIM item is scored on a 7-point Likert scale, where 1 indicates complete dependence and 7 represents complete independence. The total score ranges from 18 (complete dependence on all items) to 126 (complete independence assessed in all domains). Two sub-scores can be assessed: FIM motor (range 18–91) and FIM cognitive (range 0–35). All the evaluations were performed by certified health care professionals.

### Statistical analysis

Descriptive statistics for individual TARPP-Q items and discrete variables are reported as numbers (percentage frequency).

To investigate the existence of an underlying structure in the interrelationships among the items of the questionnaire, exploratory factor analysis was carried out.

The principal factor method was used to extract factors, followed by orthogonal rotation (Varimax method) to assist in the interpretation of the factors and to ensure that they were uncorrelated.

The determination of the number of factors to extract was guided by theory (variance explained by each factor) and seeing which number of factors yielded theoretical meaningfulness and the most interpretable results. The determination as to what the underlying factor might represent was inferred from the variables significantly loaded on their factors. An absolute factor loading value ≥ 0.35 was considered to indicate that a variable contributed significantly to a factor.

The following were verified: at least three variables (items) had to load significantly on each retained factor, the variables that loaded on a given factor needed to share conceptual meaning, and the variables that loaded on different factors considered different constructs.

Internal consistency of the factors measured in the questionnaire was assessed using Cronbach’s alpha.

The association between TARPP-Q factor scores (values at admission, at discharge, and the difference between values at discharge and admission) and age, education, and FIM total and sub-score, were analyzed by correlation analysis (Spearman r).

As to the questionnaire application, within-group comparisons were carried out by the Wilcoxon signed-rank test.

All statistical tests were two-tailed and statistical significance was set at p < 0.01. All analyses were carried out using the SAS/STAT statistical package, release 9.4 (SAS Institute Inc., Cary, NC, U.S.A.).

## Results

### Questionnaire development

A final consensus was obtained on a 31-item TARPP-Q provisional questionnaire, constituted of 10 main questions, of which 5 with multiple answers. The TARPP-Q response to each item was rated using a four-level Likert-type scale (forced choice, no neutral option): strongly disagree (score 1), disagree (score 2), agree (score 3), strongly agree (score 4).

### Questionnaire application

Forty-four patients were included in the study. Demographical and clinical data are reported in Table 1.

**Table 1:** Demographic and clinical variables

Variable	
Age (yrs)	60.7 ± 12.6
Males (%)	25 (57)
Education	10.3 ± 4.7
Disease duration	7.7 ± 9.2
Armeo–Lokomat (%)	19–25 (43–57)
Stroke (%)	16 (36)
Parkinson’s disease (%)	12 (27)
Multiple sclerosis (%)	8 (18)
Spinal cord injury (%)	4 (9)
Other* (%)	4 (9)

*Ataxia, peripheral neuropathy

All patients answered the questionnaire autonomously. There were no unaccounted answers

The responses to individual TARPP-Q items at T0 and T1 are reported in Table 2. For the sake of completeness, the mean values ± SD of all the items are also reported.

**Table 2:** TARV-Q single item responses at T0 and T1

	TARV-Q final items	Item responses at T0					Item responses at T1				
		V = 1	V = 2	V = 3	V = 4	Mean ± SD	V = 1	V = 2	V = 3	V = 4	Mean ± SD
1	It was easy to understand the exercise as requested by the device	1	3	16	24	3.43 ± 0.73	1	1	7	35	3.73 ± 0.62
2	It was easy to exercise with the device	1	5	18	20	3.30 ± 0.76	0	3	10	31	3.64 ± 0.61
3	I enjoyed exercising with the device	2	1	13	28	3.52 ± 0.76	0	1	6	37	3.82 ± 0.45
4	Movements (walking, use of arm) improved with the device	2	11	21	10	2.89 ± 0.81	0	6	24	14	3.18 ± 0.66
5A	While exercising with the device, I felt: comfortable	0	5	16	23	3.41 ± 0.69	0	1	11	32	3.70 ± 0.51
5B	While exercising with the device, I felt: uncomfortable	40	3	0	1	1.14 ± 0.51	44	0	0	0	1.00 ± 0.00
5C	While exercising with the device, I felt: clumsy	30	10	4	0	1.41 ± 0.66	35	7	2	0	1.25 ± 0.53
5D	While exercising with the device, I felt: amused	4	9	19	12	2.89 ± 0.92	5	4	15	20	3.14 ± 1.00
5E	While exercising with the device, I felt: awkward	39	4	1	0	1.14 ± 0.41	43	1	0	0	1.02 ± 0.15
5F	While exercising with the device, I felt: stressed	40	3	1	0	1.11 ± 0.39	40	3	0	1	1.14 ± 0.51
6A	While exercising with the device, I experienced: discomfort	37	4	3	0	1.23 ± 0.57	40	3	1	0	1.11 ± 0.39
6B	While exercising with the device, I experienced: well-being	5	14	18	7	2.61 ± 0.89	2	8	16	18	3.14 ± 0.88
6C	While exercising with the device, I experienced: fatigue	28	9	6	1	1.55 ± 0.82	28	10	5	1	1.52 ± 0.79
6D	While exercising with the device, I experienced: poor control of my movements	31	6	6	1	1.48 ± 0.82	36	4	3	1	1.30 ± 0.70
6E	While exercising with the device, I experienced: better control of my movements	6	8	19	11	2.80 ± 0.98	3	10	15	16	3.00 ± 0.94
7A	While exercising with the objects on the screen (Virtual Reality), I felt: comfortable	1	8	19	16	3.14 ± 0.80	1	1	19	23	3.45 ± 0.66
7B	While exercising with the objects on the screen (Virtual Reality), I felt: uncomfortable	38	5	1	0	1.16 ± 0.43	44	0	0	0	1.00 ± 0.00
7C	While exercising with the objects on the screen (Virtual Reality), I felt: clumsy	31	8	5	0	1.41 ± 0.69	36	4	3	1	1.30 ± 0.70
7D	While exercising with the objects on the screen (Virtual Reality), I felt: amused	5	7	14	18	3.02 ± 1.02	4	2	15	23	3.30 ± 0.93
7E	While exercising with the objects on the screen (Virtual Reality), I felt: awkward	39	5	0	0	1.11 ± 0.32	43	0	1	0	1.05 ± 0.30
7F	While exercising with the objects on the screen (Virtual Reality), I felt: stressed	38	5	1	0	1.16 ± 0.43	38	5	1	0	1.16 ± 0.43
8A	Seeing the score reached on the screen: makes me feel more engaged	6	15	12	11	2.64 ± 1.01	7	9	15	13	2.77 ± 1.05
8B	Seeing the score reached on the screen: makes me feel under pressure	34	6	4	0	1.32 ± 0.64	36	6	2	0	1.23 ± 0.52
8C	Seeing the score reached on the screen: aids me	15	13	10	6	2.16 ± 1.06	17	13	7	7	2.09 ± 1.10
8D	Seeing the score reached on the screen: limits me	43	1	0	0	1.02 ± 0.15	42	2	0	0	1.05 ± 0.21
8E	Seeing the score reached on the screen: makes me feel inadequate	40	4	0	0	1.09 ± 0.29	43	0	1	0	1.05 ± 0.30
9	Today, I am eager to exercise with the device	1	7	19	17	3.18 ± 0.79	0	5	16	23	3.41 ± 0.69
10A	How would you describe your experience with the device to friends / relatives? With enthusiasm	0	11	14	19	3.18 ± 0.81	0	7	15	22	3.34 ± 0.75
10B	How would you describe your experience with the device to friends / relatives? Eager to return	0	9	18	17	3.18 ± 0.76	0	3	16	25	3.50 ± 0.63
	*Removed items*										
	Instructions given by the physiotherapist were useful	0	0	12	32	3.73 ± 0.45	0	1	3	40	3.89 ± 0.39
	How would you describe your experience with the device to friends / relatives? Unsatisfied	42	0	0	2	1.14 ± 0.63	42	0	1	1	1.11 ± 0.54

After the analyses, two items (“How would you describe your experience with the device to friends/relatives? Unsatisfied” and “Instructions given by the physiotherapist were useful”) were excluded from the ultimate version of the questionnaire. Specifically, the first item did not load to any of the four factors, then was removed due to its construct inconsistency. The second one was deleted as diverting the attention from the primary interest of the questionnaire—that is, specifically, the patient-device interaction—by introducing another variable, such as the operator’s role. The authors became aware of the inconsistency of this item within the construct coherence of the questionnaire during the patient’s assessment phase only, therefore with a unanimous agreement the item was removed from the questionnaire.

The final version of the questionnaire consisted of 10 questions, 5 with multiple answers, totaling 29 items.

At the factor analysis, four factors emerged, which were named as follows, after a further consensus amongst the members of the multidisciplinary team: Factor 1: Positive attitude, Factor 2: Usability, Factor 3: Hindrance perception, and Factor 4: Distress. The values of Cronbach’s alpha ranged from a moderate 0.72 (“Distress”, Factor 4) to a high 0.92 (“Positive attitude”, Factor 1). In Table 3 items’ analysis of the TARPP-Q is reported. Cronbach’s alpha variations, obtained by removing single items from the factor, are reported in rows for each item. When appropriate, reverse scoring was applied to selected items before their inclusion in the pertaining domain.

**Table 3:** Items and factors of TARV-Q

	TARV-Q items	Factor 1	Factor 2	Factor 3	Factor 4	Cronbach alpha (single item removed)
	*Factor 1—positive attitude (variance explained = 51%; Cronbach Alpha = 0.92)*					
5A	While exercising with the device, I felt: comfortable	43	39	− 39	− 11	0.93
5D	While exercising with the device, I felt: amused	69	17	− 7	16	0.92
6B	While exercising with the device, I experienced: well-being	36	27	− 7	− 20	0.93
7A	While exercising with the objects on the screen (Virtual Reality), I felt: comfortable	82	20	− 18	− 23	0.91
7D	While exercising with the objects on the screen (Virtual Reality), I felt: amused	80	18	− 10	− 17	0.91
8A	Seeing the score reached on the screen: makes me feel more engaged	68	4	18	− 36	0.92
8C	Seeing the score reached on the screen: aids me	66	− 9	25	− 45	0.92
9	Today, I am eager to exercise with the device	89	17	− 18	− 1	0.91
10B	How would you describe your experience with the device to friends / relatives? Eager to return	88	10	− 12	− 6	0.91
10A	How would you describe your experience with the device to friends / relatives? With enthusiasm	84	10	− 17	− 16	0.91
	*Factor 2—usability (variance explained = 21%; Cronbach Alpha = 0.84)*					
1	It was easy to understand the exercise as requested by the device	12	71	− 18	9	0.81
2	It was easy to exercise with the device	16	83	− 14	− 9	0.77
3	I enjoyed exercising with the device	10	62	13	6	0.81
4	Movements (walking, use of arm) improved with the device	31	57	8	12	0.81
6E	While exercising with the device, I experienced: better control of my movements	43	56	− 20	− 12	0.85
	*Factor 3—perception of hindrance (variance explained = 15%; Cronbach Alpha = 0.73)*					
5B	While exercising with the device, I felt: uncomfortable	− 35	− 6	67	− 10	0.63
5E	While exercising with the device, I felt: awkward	5	10	58	4	0.72
6D	While exercising with the device, I experienced: poor control of my movements	35	− 36	40	25	0.75
7B	While exercising with the objects on the screen (Virtual Reality), I felt: uncomfortable	− 2	− 4	45	− 2	0.71
7E	While exercising with the objects on the screen (Virtual Reality), I felt: awkward	− 17	− 5	90	23	0.60
	*Factor 4—distress (variance explained = 13%; Cronbach coefficient alpha = 0.72)*					
5C	While exercising with the device, I felt: clumsy	33	− 39	27	44	0.70
5F	While exercising with the device, I felt: stressed	− 18	15	6	42	0.72
6A	While exercising with the device, I experienced: discomfort	− 18	− 18	− 5	39	0.69
6C	While exercising with the device, I experienced: fatigue	0	− 43	− 6	41	0.69
7C	While exercising with the objects on the screen (Virtual Reality), I felt: clumsy	− 8	− 27	− 6	59	0.67
7F	While exercising with the objects on the screen (Virtual Reality), I felt: stressed	− 23	10	2	53	0.69
8B	Seeing the score reached on the screen: makes me feel under pressure	− 18	− 39	9	34	0.68
8D	Seeing the score reached on the screen: limits me	11	− 41	− 5	25	0.71
8E	Seeing the score reached on the screen: makes me feel inadequate	− 2	2	17	45	0.69
	*Items excluded*					
	How would you describe your experience with the device to friends / relatives? Unsatisfied	27	12	15	− 14	
	Instructions given by the physiotherapist were useful	26	35	3	− 5	

Values extracted for the Factors are multiplied by 100 and rounded to the nearest integer

Cronbach’s alpha values obtained removing single items from the factor, are reported in the last column, in rows for each item

In Additional file 1 printable English version is available.

In Additional file 2 printable Original Italian version is available.

Descriptive statistics for the total TARPP-Q score and the FIM (motor, cognitive and total) at T0 and T1 and the 4 factors are reported in Table 4.

**Table 4:** Descriptive statistics for the 4 factors, the total TARPP-Q and FIM scores at T0 and T1

	Mean ± SD T0	Mean ± SD T1	Delta T1-T0	p val
*TARV-Q factors and total scores*				
Positive attitude	29.41 ± 6.72	31.84 ± 6.10	2.43 ± 3.84	0.00026
Usability	15.93 ± 3.16	17.36 ± 2.10	1.43 ± 1.96	< 0.0001
Hindrance perception	6.02 ± 1.70	5.36 ± 0.78	− 0.66 ± 1.71	0.016
Distress	11.30 ± 2.68	10.80 ± 2.27	− 0.50 ± 1.95	0.11
TARV-Q total score	62.66 ± 7.91	65.36 ± 7.22	2.70 ± 4.64	0.0008
*FIM scores*				
FIM cognitive	32.02 ± 2.63	32.34 ± 2.63	0.32 ± 0.86	0.016
FIM motor	64.43 ± 20.75	73.82 ± 15.09	9.39 ± 11.84	< 0.0001
FIM total score	96.48 ± 22.21	105.89 ± 16.27	9.41 ± 11.96	< 0.0001

p val: < 0.01 Wilcoxon signed rank test

Factor’s score range: Positive attitude (T0 = 16–40, T1 = 16–40); Usability (T0 = 7–20, T1 = 12–20); Hindrance perception (T0 = 5–12, T1 = 5–8); Distress (T0 = 9–19, T1 = 9–19)

TARPP-Q score range (T0 = 46–80, T1 = 49–78)

FIM ranges: FIM Cognitive (T0 = 25–35, T1 = 26–35); FIM Motor (T0 = 13–89, T1 = 35–91); FIM Total score (T0 = 44–124, T1 = 65–125)

FIM scores and all TARPP-Q factors, except for Distress (p = 0.11), significantly improved at the end of the rehabilitation treatment.

The association between age, education and FIM scores, and TARPP-Q factors were also assessed, but no significant relationship was observed with any domain at T0 nor T1. Analogously, no significant association was observed between age, education, and changes (values at T1 – values a T0) in FIM scores vs changes in TARPP-Q factors.

Table 5 reports the correlation analysis between factors.

**Table 5:** Correlations between factors

	Positive attitude	Usability	Hindrance perception	Distress
Positive attitude		0.53^‡^	0.03	− 0.33^^^
Usability	0.53^‡^		− 0.27	− 0.37^^^
Hindrance perception	0.03	− 0.27		0.30^^^
Distress	− 0.33^^^	− 0.37^^^	0.30^^^	

^p < 0.05; ^†^p < 0.01; ^‡^p < 0.001

## Discussion

The assessment of high-technology devices according to patients’ perspectives is a relevant—and relatively young—topic for motor rehabilitation [2, 3]. Research studies proved that non-motor variables such as patient positive disposition, perception of safety, motivation, or engagement may have significant positive effects on the efficacy of technology-assisted training [2, 13, 16, 33]. However, it is currently under debate whether the use of high technology or robotic devices may also convey negative feelings, being felt by users as uncomfortable, disorienting, or even a source of fear [34].

The TARPP-Q was conceived to cover different facets of patients’ experiences identified through a Delphi methodology by a multidisciplinary team. Relevant determinants such as Positive attitude, Usability, Hindrance perception, and Distress were identified. Since most of the currently available high-technology devices are integrated with exergames [9], pertaining questions were also enclosed in the item set.

### Questionnaire development

Overall, the TARPP-Q showed sound psychometric properties. Four factors were identified by an exploratory factor analysis, which resulted in coherence with clinical experience (construct validity) and proved to have a good internal consistency (sound values of total Cronbach’s alpha and of Cronbach’s alpha variations, obtained by removing single items from the factor).

*Positive Attitude (Factor 1)*. The first factor had high internal consistency (Cronbach Coefficient Alpha = 0.92) and included items related to positivity, amusement, comfort, aid, and engagement. Given the nature of the item aggregation, the factor was labeled as “Positive attitude”—referring to patients’ positive set of emotions, beliefs, and behaviors toward the technology-assisted device. Although structural and ergonomic characteristics of devices are indeed drivers of treatment efficacy [35] and safety [14], patients’ Positive attitude could positively impact the device constant usage, preventing discontinuations and drop-offs. Literature findings suggested how personality traits and beliefs may also influence change-promotion behaviors while using devices [36, 37]; moreover, a sense of comfort (items 5A and 7A) is a prerequisite for a safe application of technology-assisted devices over time [14] and primary requirement for a stable motor recovery [38, 39]. In addition, amusement (items 5D and 7D) and enthusiasm (item 10A) also affect rehabilitation outcomes, particularly when related to the use of exergames [9, 40]. Similarly, engagement (items 8A, 9, and 10B) and well-being (item 6B) are known to impact the training, even if mediated by the type of training or device (repetition and variation) [41, 42] and by patient expectations [25]. Lastly, patient perception of aid (item 8C) while performing exercises is only partially covered in literature and deserved further investigation [43].

*Usability (Factor 2)*. Usability can be defined as the capacity of a system to allow users to perform tasks safely, effectively, and efficiently while enjoying the experience [44]. Assessing usability in rehabilitation ensures device maximum functionality, whilst increasing effectiveness, engagement, and ease of learning. As a result, the usability of TAR devices has been widely investigated, with encouraging results as to patients and health care professionals’ device perception and execution of movements that are accurate, natural, and harmless for the patient [25, 44, 45]. Items about Usability emerged as a factor in TARPP-Q*,* showing a sound internal consistency (Cronbach Coefficient Alpha = 0.84) and collecting items investigating ease and enjoyableness of use. Interestingly, two items related to perceived performance improvement also loaded to Usability (4, 6E). On this note*,* is it worth noticing how a user-friendly device leads to positive perception (items 1, 2, 3), helping movement execution (items 4 and 6E) in a way to influence treatment motor outcomes and exergames scores [9, 46, 47].

*Hindrance perception (Factor 3)*. The third factor found (Cronbach Coefficient Alpha = 0.73) collects both negative implications on motor execution of exercises (i.e. poor movement control; item 6D) and psychological facets that may interfere with device usage, such as feeling awkward (items 5E and 7E) and being uncomfortable (items 5B and 7B). In the current context, Hindrance perception is to be referred to as subjective sensations (constraint, impediment) deriving directly from “wearing” the exoskeleton while performing exercises, as already expressed in other works [48, 49]. It may be also inferred that “feeling” the device guiding movements and enveloping body parts might be perceived as something out of ordinary, determining a bizarre experience. Indeed, the present distinction deserves further clarification, especially regarding the subjective experience of motor control and body constraint. The emerged meaningful concept, however, witness the multi-level complexity of TAR [38], encouraging the adoption of a holistic perspective to treatment [25, 42]. Negative perceptions towards technological devices are indeed a wake-up call for health care professionals, as they might considerably interfere with motivation and result in treatment discontinuity or rejection [50].

*Distress (Factor 4)*. Distress can be defined as a state of emotional suffering associated with stressors and demands that are difficult to cope with in daily life [51]. As a barrier to technology-assisted devices, Distress emerged as the fourth factor, showing a moderate internal consistency (Cronbach Coefficient Alpha = 0.72). Recent studies suggested conducting an in-depth analysis of the relationship between high-technology or robotic devices and psychological responses [52]. Consistently, assessing negative feelings and psychological domains could provide considerations of clinical interest (items 5F, 7F, 8B, 8D). In this regard, given the exploratory factor analysis, exergaming-items 8B (“Seeing the score reached on the screen: makes me feel under pressure”) and 8D (“Seeing the score reached on the screen: limits me”) (“Distress”) have been included to Factor 4, to further characterize the distinction emerged during the analyses: Factor 4 (Distress), mostly focusing on psychological shades of performance limitations and distress, versus awkwardness and physical sensation of impediment, pertaining Factor 3 (Hindrance perception).

Accordingly, items 8E (“makes me feel inadequate”), 5C, 7C (“clumsy”) and 6A (“discomfort”) evaluate psychological facets of a patient’s perception of TAR. However, further studies are needed to systematically investigate the role of specific psychological traits (such as self-esteem) and emotional statuses during the treatment by using tailored instruments [10, 33, 36, 42].

Finally, factor analysis showed that item 6C “fatigue” (“While exercising with the device, I experienced: fatigue”) loaded to Factor 4 (Distress). Fatigue is a central issue for motor rehabilitation, particularly in neurologic diseases such as Parkinson’s disease [53], multiple sclerosis [54], and stroke [55], and prominently affects treatment motivation and rehabilitation efficacy [56]. However, in the field of rehabilitation, fatigue, both in its definition and etiology, is still under debate. For this reason, the item “Fatigue”, which resulted included in Distress—and thus in a psychological factor—could be regarded as such for its psychological implications only. Future studies on fatigue together with large-scale applications of TARPP-Q may better clarify its role.

### Questionnaire application

As to the questionnaire application, statistical analysis among the TARPP-Q factors, demographic, and clinical variables such as age, education, and patients’ disability level (expressed by FIM score at T0 and T1), identified no significant correlations. This result suggests that, regardless of age [15], socio-cultural context, and level of disability, patient technology acceptance is largely positive in our sample. In this respect, the age of the patient, for example, might be a conditioning factor in the acceptance of TAR [15]. In previous work, similar results were obtained by using the PIADS scale [24].

The four factors’ pre-post training changes (Table 5), obtained during the questionnaire application, further clarify this latter consideration: while FIM scores (Motor and Total subscales), Positive attitude, Usability, and Hindrance perception significantly improved at T1. Distress only remained unchanged after training.

The significant increase in Positive attitude and Usability factors is probably due to progressive experience determined by the training itself, whilst enhancing patient competence [41, 57]. As for the improvement of Positive Attitude, it is well known that patient perception of rehabilitation devices is associated with treatment satisfaction and not exclusively with physical improvement [10, 16]. Overall, results confirm the role of Positive attitude and Usability, while highlighting that patients’ psychological traits are crucial determinants in the interaction with a technology-assisted device [37]. Notably, in our sample, Usability resulted significantly correlated to patient Positive Attitude, proving the pivotal role of patient positive feelings towards the perceived usability of TAR.

Concerning both the factors Hindrance perception and Distress**,** the frequency distribution of TARPP-Q single item responses at T0 (Table 2) showed low levels of distress and negative perception. Consequently, fear and skepticism towards technology-assisted devices [34] resulted to be low or at least controlled in our sample. This might be due to different reasons, ranging from effective patient-healthcare professional communication to the personal characteristics of the participants. Future studies may furtherly investigate this theme, suggesting best practices to empower patients’ disposition during the interaction with TAR devices.

Interestingly, at present, factor analysis showed that items with a negative meaningful concept aggregate into two separate factors (Factor 3, Hindrance perception; Factor 4, Distress). Given the overall similarity of the contents, further studies are needed to better define the nature of the theoretical differences between these two factors.

As to patients’ perception of movement (6D. Poor control of my movements; 6E. Better control of my movements; 4. Movements (walking, use of arm) improved with the device), the TARPP-Q highlighted a betterment of both motor control and functionality. Indeed, clinical implications of movement control in TAR are noteworthy and still partially to be investigated [44, 45].

Finally, concerning the exergaming section of the questionnaire, factor analysis showed that pertaining items were distributed in separate factors (Factor 1, Factor 3, Factor 4). The non-aggregation may suggest that the role of exergaming in neurorehabilitation is multidimensional and still to be clarified, as a result of the complex combination of both motor and cognitive effects [58–60].

### Limitations and future developments

TARPP-Q development was exclusively guided by collecting theoretical inputs from empirical observation and clinical experience. Nevertheless, the questionnaire aims to collect information from a relatively novel field [3, 18], an assumption that account for the exploratory nature of the study. The questionnaire, however, was administered in expert-lead settings, consisting of an interprofessional board of specialists in neurology, physiatry, physiotherapy, psychology, and bioengineering, whose strict and ongoing confrontation—together with the implementation of the Delphi procedure—may have strengthened its reliability.

It is important to highlight that the present results derive from the self-evaluation of patients who have carried out rehabilitation exclusively with fixed exoskeletons. Future studies may ascertain if similar considerations could be extended to other technologies (wearable, portable devices, etc.) which perform as motor “aids” to patients’ body parts during natural gait or upper-limb movements. Also, further studies are needed to determine the temporal stability of the TARPP-Q and to better define the nature of the two “negative” factors (Factor 3, Factor 4).

Given the multiple facets of exergaming and rehabilitation, future refinements of the TARPP-Q may consider extending the number of items dedicated to the theme (including a more structured reference to Virtual Reality and Augmented Reality), to provide a fine-grained analysis of the phenomenon [60, 61].

As to the pilot study, the main limitation is represented by the small sample, mainly composed of older people. However, it is well known that studies on this topic often consider a small number of patients. Indeed, a strength of the questionnaire relies on its multidisciplinary, as assessing general features of technology-assisted devices and exergames perception to describe both motor and emotional implications of patient-device interaction through a combination of determinants such as device usability, motor control, attitude, fatigue, and distress.

Lastly, the questionnaire was administered to patients who spoke Italian only and still needs full validation in terms of reliability, validity, and sensitivity. Rigorous validation of the TARPP-Q in the English language will be the object of a second study, possibly involving an international consortium so to allow comparison of validation results. Future developments of this questionnaire might also help shed light on the different nuances of meaning that might have been “lost in translation” during the Italian-English transposition.

## Conclusions

The TARPP-Q aims to describe patients’ experience with Technology-Assisted Rehabilitation and exergaming. Results showed the role of four factors (Positive attitude, Usability, Hindrance perception, Distress) related to the direct patient experience with the device. Particularly, Usability is a pivotal parameter for patient performance in rehabilitation and it is directly correlated with patient Positive Attitude. Age, education, and disability level are not conditioning factors for patient experience with TAR.

Large-scale applications and full validation of the present questionnaire may clarify how patient perception affects training effectiveness, helping to customize device settings according to patients’ characteristics. It might be also suggestable to test the TARPP-Q in different clinical populations and across different TAR devices using exergaming.

Finally, the present contribution may hopefully help direct future investigations towards the often-unaccounted effect of patients’ psychological concerns [62], as the role of motor recovery and functional outcome largely prevail in the current research [39, 63]. Further investigations are needed to account for both those relevant facets—strongly bonded—and across different technology-assisted devices.

## Supplementary Information

**Additional file 1.** TARPP-Q Questionnaire (English translation).

**Additional file 2.** TARPP-Q Questionnaire (Original Italian version).

## Data Availability

Data and materials are available if requested.

## Abbreviations

TARPP-Q: Technology assisted rehabilitation patient perception questionnaire
TAR: Technology-assisted rehabilitation
APF: Augmented performance feedback
FIM: Functional independence measure
MMSE: Mini Mental State Examination
PIADS: Psychosocial Impact of Assistive Device Scale
