# Supplementary material for: Technology Assisted Rehabilitation Patient Perception Questionnaire (TARPP-Q): development and implementation of an instrument to evaluate patients’ perception during training
Source: J Neuroeng Rehabil. 2023 Mar 24;20:35. doi: 10.1186/s12984-023-01146-3 (PMC10037786; doi:10.1186/s12984-023-01146-3)
Supplement: Supplementary file 1 — Additional file 1. TARPP-Q Questionnaire (English translation). [file 12984_2023_1146_MOESM1_ESM.pdf]

TARPP-Q
(Technology-Assisted Rehabilitation Patient Perception Questionnaire - TARPP-Q)

The questionnaire was developed to verify the degree of patient appreciation and engagement when assisted by a technological/robotic device for rehabilitation. There are no right or wrong answers; your opinion is very valuable for us.

	STRONGLY DISAGREE	MILDLY DISAGREE	MILDLY AGREE	STRONGLY AGREE
**1. It was easy to understand the exercise as requested by the device**	1	2	3	4
**2. It was easy to exercise with the device**	1	2	3	4
**3. I enjoyed exercising with the device**	1	2	3	4
**4. Movements (walking, use of arm) improved with the device**	1	2	3	4

**5. While exercising with the device, I felt:**	STRONGLY DISAGREE	MILDLY DISAGREE	MILDLY AGREE	STRONGLY AGREE
5A. Comfortable	1	2	3	4
5B. Uncomfortable	1	2	3	4
5C. Clumsy	1	2	3	4
5D. Amused	1	2	3	4
5E. Awkward	1	2	3	4
5F. Stressed	1	2	3	4
**6. While exercising with the device, I experienced:**	STRONGLY DISAGREE	MILDLY DISAGREE	MILDLY AGREE	STRONGLY AGREE
6A. Discomfort	1	2	3	4
6B. Well-being	1	2	3	4
6C. Fatigue	1	2	3	4
6D. Poor control of my movements	1	2	3	4
6E. Better control of my movements	1	2	3	4

**7. While exercising with the objects on the screen (exergames), I felt:**	STRONGLY DISAGREE	MILDLY DISAGREE	MILDLY AGREE	STRONGLY DISAGREE
7A. Comfortable	1	2	3	4
7B. Uncomfortable	1	2	3	4
7C. Clumsy	1	2	3	4
7D. Amused	1	2	3	4
7E. Awkward	1	2	3	4
7F. Stressed	1	2	3	4
**8. Seeing the score reached on the screen (exergames):**	STRONGLY DISAGREE	MILDLY DISAGREE	MILDLY AGREE	STRONGLY AGREE
8A. Makes me feel more engaged	1	2	3	4
8B. Makes me feel under pressure	1	2	3	4
8C. Aids me	1	2	3	4
8D. Limits me	1	2	3	4
8E. Makes me feel inadequate	1	2	3	4

	STRONGLY DISAGREE	MILDLY DISAGREE	MILDLY AGREE	STRONGLY AGREE
**9. Today, I am eager to exercise with the device**	1	2	3	4
**10. How would you describe your experience with the device to friends / relatives?**	STRONGLY DISAGREE	MILDLY DISAGREE	MILDLY AGREE	STRONGLY AGREE
10A. With enthusiasm	1	2	3	4
10B. Eager to return	1	2	3	4

Edited by: Fundarò C, Giardini A, Salvini S, Traversoni S; Colombo R., Casale R. - Istituti Clinici e Scientifici Maugeri, IRCCS Montescano (PV); Istituti Clinici e Scientifici Maugeri, IRCCS Pavia (PV); Casa di Cura Habilita Spa, Zingonia di Ciserano (BG).
