# Supplementary material for: Technology Assisted Rehabilitation Patient Perception Questionnaire (TARPP-Q): development and implementation of an instrument to evaluate patients’ perception during training
Source: J Neuroeng Rehabil. 2023 Mar 24;20:35. doi: 10.1186/s12984-023-01146-3 (PMC10037786; doi:10.1186/s12984-023-01146-3)
Supplement: Supplementary file 2 — Additional file 2. TARPP-Q Questionnaire (Original Italian version). [file 12984_2023_1146_MOESM2_ESM.pdf]

TARPP-Q
(Technology-Assisted Rehabilitation Patient Perception Questionnaire - TARPP-Q)

Istruzioni: Il presente questionario viene somministrato per verificare il grado di apprezzamento e di coinvolgimento del paziente trattato con un dispositivo riabilitativo. Non ci sono risposte giuste o sbagliate; per noi è importante conoscere la sua opinione.

	PER NULLA	UN PO'	ABBASTANZA	MOLTO
**1. E' stato facile capire il compito richiesto dal dispositivo**	1	2	3	4
**2. E' stato facile eseguire gli esercizi con il dispositivo**	1	2	3	4
**3. L'uso del dispositivo mi è piaciuto**	1	2	3	4
**4. Il movimento (cammino, uso del braccio) è migliorato con il dispositivo**	1	2	3	4

**5. Nel lavorare con il dispositivo robotico mi sono sentito:**	**PER NULLA**	**UN PO'**	**ABBASTANZA**	**MOLTO**
5A. A mio agio	1	2	3	4
5B. A disagio	1	2	3	4
5C. Impacciato	1	2	3	4
5D. Divertito	1	2	3	4
5E. Imbarazzato	1	2	3	4
5F. Stressato	1	2	3	4
**6. Nel lavorare con il dispositivo robotico ho provato:**	**PER NULLA**	**UN PO'**	**ABBASTANZA**	**MOLTO**
6A. Fastidio	1	2	3	4
6B. Benessere	1	2	3	4
6D. Fatica	1	2	3	4
6E. Poco controllo sui miei movimenti	1	2	3	4
6F. Maggior controllo sui miei movimenti	1	2	3	4

**7. Nel lavorare con le cose viste sullo schermo (Realtà Virtuale) mi sono sentito:**	**PER NULLA**	**UN PO'**	**ABBASTANZA**	**MOLTO**
7A. A mio agio	1	2	3	4
7B. A disagio	1	2	3	4
7C. Impacciato	1	2	3	4
7D. Divertito	1	2	3	4
7E. Imbarazzato	1	2	3	4
7F. Stressato	1	2	3	4
**8. La possibilità di vedere il punteggio raggiunto durante l'esercizio:**	**PER NULLA**	**UN PO'**	**ABBASTANZA**	**MOLTO**
8A. Mi stimola ancora di più	1	2	3	4
8B. Mi fa sentire sotto pressione	1	2	3	4
8C. Mi agevola	1	2	3	4
8D. Mi ostacola	1	2	3	4
8E. Mi fa sentire inadeguato	1	2	3	4

	PER NULLA	UN PO'	ABBASTANZA	MOLTO
**9. Oggi ho voglia di venire a fare riabilitazione con il dispositivo:**	1	2	3	4
**10. Come descriveresti ai tuoi amici/familiari quest'esperienza?**	**PER NULLA**	**UN PO'**	**ABBASTANZA**	**MOLTO**
10A. Con entusiasmo	1	2	3	4
10B. Con desiderio di ritornare	1	2	3	4

A cura di: Fundarò C, Giardini A, Salvini S, Traversoni S. Istituti Clinici e Scientifici Maugeri, IRCCS Montescano (PV); Colombo R, Istituti Clinici e Scientifici Maugeri, IRCCS Pavia (PV). Casale R. Casa di Cura Habilita Spa, Zingonia di Ciserano (BG).
